# The impact of disease-related symptoms and palliative care concerns on health-related quality of life in multiple myeloma: a multi-centre study

**DOI:** 10.1186/s12885-016-2410-2

**Published:** 2016-07-07

**Authors:** Christina Ramsenthaler, Thomas R. Osborne, Wei Gao, Richard J. Siegert, Polly M. Edmonds, Stephen A. Schey, Irene J. Higginson

**Affiliations:** Department of Palliative Care, Policy and Rehabilitation, Cicely Saunders Institute, King’s College London, School of Medicine, Bessemer Road, London, SE5 9PJ UK; Auckland University of Technology, Auckland, New Zealand; Department of Palliative Care, King’s College Hospital NHS Foundation Trust, London, UK; Department of Haematological Medicine, King’s College Hospital NHS Foundation Trust, London, UK

**Keywords:** Multiple myeloma, Health-related quality of life, Palliative Care Outcome Scale, Symptom burden, Quality of life, Palliative care

## Abstract

**Background:**

Multiple myeloma, the second most common haematological cancer, remains incurable. Its incidence is rising due to population ageing. Despite the impact of the disease and its treatment, not much is known on who is most in need of supportive and palliative care.

This study aimed to (a) assess symptom severity, palliative care concerns and health-related quality of life (HRQOL) in patients with multiple myeloma, and (b) to determine which factors are associated with a lower quality of life. We further wanted to know (c) whether general symptom level has a stronger influence on HRQOL than disease characteristics.

**Methods:**

This multi-centre cross-sectional study sampled two cohorts of patients with multiple myeloma from 18 haematological cancer centres in the UK. The Myeloma Patient Outcome Scale (MyPOS) was used to measure symptoms and concerns. Measures of quality of life included the EORTC QLQ-C30, its myeloma module and the EuroQoL EQ-5D. Data were collected on socio-demographic, disease and treatment characteristics and phase of illness. Point prevalence of symptoms and concerns was determined. Multiple regression models quantified relationships between independent factors and the MyPOS, EORTC global quality of life item and EQ5D Index.

**Results:**

Five-hundred-fifty-seven patients, on average 3.5 years (SD: 3.4) post-diagnosis, were recruited. 18.2 % had newly diagnosed disease, 47.9 % were in a treatment-free interval and 32.7 % had relapsed/progressive disease phase. Patients reported a mean of 7.2 symptoms (SD: 3.3) out of 15 potential symptoms. The most common symptoms were pain (72 %), fatigue (88 %) and breathlessness (61 %). Those with relapsed/progressive disease reported the highest mean number of symptoms and the highest overall palliative care concerns (*F* = 9.56, *p* < 0.001). Factors associated with high palliative care concerns were a general high symptom level, presence of pain, anxiety, low physical function, younger age, and being in the advanced stages of disease.

**Conclusion:**

Patients with multiple myeloma have a high symptom burden and low HRQOL, in the advanced and the earlier stages of disease. Identification of patients in need of supportive care should focus on assessing patient-reported outcomes such as symptoms and functioning regularly in clinical practice, complementary to traditional biomedical markers.

**Electronic supplementary material:**

The online version of this article (doi:10.1186/s12885-016-2410-2) contains supplementary material, which is available to authorized users.

## Background

Haematological malignancies belong to the most common cancers worldwide [1]. Multiple myeloma is the second most common haematological malignancy with an incidence of 3.29 to 4.82 per 100,000 individuals per year worldwide [2]. Multiple myeloma is characterised by a specific pattern of end-organ damage with destruction of the bones, bone marrow failure and renal failure. With the introduction of novel therapies and autologous stem-cell transplantation survival has been extended, especially for patients younger than 60 years [3]. However, since multiple myeloma remains an incurable disease, life expectancy is limited. 40.3 and 20.5 % of patients survive 5 and 10 years, respectively [3, 4]. Despite improvements in therapies, patients face progressive disease, interspersed with intervals of stable disease with minimal or maintenance treatment [5]. Symptoms may persist into treatment-free intervals [6], added onto which treatment-related toxicity further impacts on health-related quality of life (HRQOL) [7, 8].

There is evidence that myeloma patients suffer more symptoms and problems than other haematological cancers. A study from Denmark reported a mean symptom level of 5.6 symptoms with 2.3 symptoms identified as severe [9]. Myeloma patients reported the highest level of pain, fatigue and constipation, alongside problems with physical, role, and social function [9, 10]. A study from the Eindhoven cancer registry including myeloma patients up to 10 years post-diagnosis and comparing results with an age- and gender-matched normative population, found similarly diminished and clinically relevant compromises in all functioning subscale scores of the EORTC QLQ-C30 questionnaire [11]. Again, symptoms of pain, fatigue, but also breathlessness, nausea and vomiting and peripheral neuropathy were reported by patients to be the most bothersome symptoms [11]. The general high symptom level and the importance of high symptom burden in conjecture with mental health symptoms were identified as strong determinants of health-related quality of life (HRQOL) in a recent study enrolling myeloma outpatients in a multi-centre, cross-sectional study [12].

Longitudinal observational evidence of how HRQOL changes over the disease course focuses entirely on stem cell transplantation populations. Here, results mainly support the fact that myeloma patients experience a high symptom burden even before stem cell collection, as shown in a study with 94 patients receiving high dose melphalan and autologous stem cell transplantation, reporting at least moderate fatigue, pain, anxiety and depression at baseline [13]. After transplantation most symptoms improved, but depression and overall quality of life deteriorated. That recovery to full functioning and symptom levels prior to therapy is often not possible for patients with myeloma was demonstrated by a cross-sectional postal survey of 650 patients at different disease stages [6]. Recovery during subsequent treatment-free intervals was often not fully achieved and patients lived with a profound impact of the disease, its disease-related symptoms but also treatment-related toxicities [6].

Thus, the disease is an example of the changing face of cancer with patients experiencing a chronic disease trajectory [14] during which a variety of symptoms, psychological and social factors impact on patients’ quality of life. However, the aspect of quality of life is still underrepresented in myeloma research, both as an outcome in evaluation of cancer treatment and in impacting treatment and supportive care guidelines [15, 16]. Descriptive studies of HRQOL are mainly cross-sectional in nature and focus on treatment or trial populations that receive autologous stem cell transplantation [17–22]. However, information on patients in later treatment phases is mainly lacking. Only one study by Boland and co-authors enrolled patients at a median of 5.5 years post diagnosis, including patients in later treatment intervals [23]. Thus, relatively little is known about how HRQOL and physical and psychosocial symptoms change over time and in the advanced stages of disease. This information would be vital to understand when patients experience periods in the disease trajectory during which they would benefit from additional support. This would help target services to those individuals most at risk, who could then benefit from early and preventive supportive care interventions. Further, the role of general symptom level and other disease- and treatment-related determinants in their influence on HRQOL remains conflicting [12, 16]. In focusing on the advanced stages of myeloma, existing and commonly used questionnaires such as the EORTC QLQ-C30 might underrepresent some of the problems and concerns regarding information and service provision that are of particular interest to myeloma patients [24]. We therefore wanted to focus on further problems and concerns that are important to patients with multiple myeloma, in addition to symptom burden, and to understand how symptom burden and problems differ during different treatment phases.

In this study we sought to determine the prevalence and severity of common symptoms and problems in patients with multiple myeloma at various stages of their disease, specifically for those with relapsed or progressive disease; and to determine whether patients in the advanced stages of myeloma experience a different symptom and problem profile than patients in earlier stages. We also sought to determine which demographic and disease characteristics were associated with a lower quality of life and more symptoms and problems, testing the hypothesis whether general symptom level and specific symptoms had a stronger influence on HRQOL than disease characteristics.

## Methods

### Study design and participants

For this multisite, cross-sectional study patients with multiple myeloma were recruited from both inpatient stem cell transplantation units and outpatient haematology clinics in 18 centres in the United Kingdom. Participating hospitals included a mixture of tertiary transplant centres and district general hospitals to ensure a representative sample of patients from different settings. The analysis for this study consists of two cohorts of patients that were recruited 1 year apart (cohort 1 was recruited from February 2013 to August 2013 and cohort 2 was recruited from April 2014 to September 2014) – one cohort for validating a new questionnaire to measure disease-specific quality of life in multiple myeloma (the Myeloma Patient Outcome Scale, MyPOS) (*n* = 380 myeloma patients) and one cohort for a longitudinal study, determining the impact of physical and mental symptoms on quality of life, and enrolling patients with multiple myeloma that were either newly diagnosed or had received treatment before (*n* = 235 myeloma patients).

Inclusion criteria for both studies were: age ≥18 years, confirmed diagnosis of multiple myeloma that had been disclosed to the patient, and the capacity to give informed written consent. Exclusion criteria were: Patients who were too unwell, distressed or symptomatic to participate as judged by their clinical team, patients with severe neutropenia or for whom myeloma was not the most important health problem.

### Procedures

Consecutive patients were screened by a member of the clinical team for eligibility before being approached by clinicians in the clinic or on the ward. If they signalled interest they then met with a research nurse who explained the study and obtained written consent. All were informed that participation was voluntary and would not affect the medical management in any way. At this point the research nurse also completed the demographic information with the participant. The patient-reported questionnaires were completed by patients in paper format either during their clinic visit or at home. In case of completion at home, patients were supplied a pre-paid envelope for returning the questionnaires to the institute. Information on patients’ medical history and the treatments they had received was extracted from the medical notes by the clinicians or research nurses with the permission of the patient. All non-participants (those who were ineligible and those who declined) were asked for consent to record limited demographic and treatment details in order to compare these against the study sample.

### Data collection and measures

#### Patient-reported outcome variables

The two main outcomes of the study, quality of life and symptom burden/palliative care concerns, were assessed using two generic and two disease-specific questionnaires. Choice of patient-reported outcomes was based on a systematic review of HRQOL validated in multiple myeloma [25]. Generic quality of life was measured with the European Organization for Research and Treatment of Cancer (EORTC) quality of life questionnaire QLQ-C30 (version 3) [26] and the EuroQOL 5D-3L questionnaire [27]. One myeloma-specific quality of life questionnaire, the EORTC QLQ-MY20 [28, 29], was used to reflect disease-specific symptoms and concerns. Both the generic and the disease-specific version of the EORTC were chosen as they have undergone the most extensive psychometric validation in myeloma patients [25], are considered to be the gold standard in clinical trials [15] and therefore give a valid account of HRQOL in multiple myeloma. Scores from the EORTC QLQ-C30 were linearly transformed and subscales were formed according to the published guidelines [30]. For the myeloma module QLQ-MY20, the two symptom subscales and two functional subscales were formed according to the guidelines published in the initial validation study [28, 29]. For the EuroQOL 5D-3L questionnaire, the US norms were used to convert the health states into the single summary index [27].

The Myeloma Patient Outcome Scale (MyPOS) [31] formed the main outcome for determining point prevalence of disease- and treatment-related symptoms and to measure palliative care concerns, such asconcerns regarding functional ability in daily life, feeling at peace, concerns regarding the future and fear of dying, information needs and concerns regarding practical matters and financial burden of disease (for all items in the questionnaire see Additional file 1: Figure S1). Palliative care concerns in this context focus on the outcomes that reflect the specific goals of palliative care, namely to promote an individual’s quality of life and to relieve any distressing symptoms and to offer emotional, spiritual and psychological support [32]. The MyPOS therefore focuses on assessing those areas that are key domains for patients experiencing a higher disease burden. The MyPOS is the only available questionnaire that assesses outcomes important to palliative care in late stage and earlier but symptomatic disease across settings [32]. The generic and disease-specific outcome measures in their combination allow to determine which myeloma patients experience a high burden, either through a high symptom level, high burden from specific symptoms or from wider psychological, spiritual or practical concerns.

The presence of clinically relevant anxiety or depression was measured using the Hospital Anxiety and Depression Scale (HADS) [33], both of which are common problems in cancer patients and might be important problems associated with burden and HRQOL. The Hospital Anxiety and Depression Scale is a validated self-report questionnaire consisting of 14 items, seven items each assessing depression or anxiety with the two subscale scores ranging from 0 to 21. A cut-off point of 8 out of 21 per subscale is used to define clinical cases of depression or anxiety, respectively, and higher scores indicate higher depression or anxiety [34].

Table 1 presents a short description of each outcome measure and its scoring procedure.

**Table 1:** Data collection and questionnaires for outcome collection

	Measure	Description
Symptom status and palliative care concerns	Myeloma Patient Outcome Scale (MyPOS) [31]	33-item questionnaire with 15 disease- and treatment-specific symptoms, 13 myeloma-specific quality of life items, 5 generic items about palliative care concerns
		Module of the Palliative Care Outcome Scale [32]
		Three subscales: Functioning and symptoms, Emotional response, Healthcare support (information and satisfaction with care) [31]
		5-point Likert scale (0 – not at all to 4 – overwhelming)
		Possible range of 0–132 for total score (higher score means more symptoms/problems)
Health-related quality of life	European Organization for Research and Treatment of Cancer (EORTC) QLQ-C30 [26]	30-item generic health-related quality of life questionnaire
		Five functional scales (physical, role, emotional, social, cognitive functioning), six symptom scales (fatigue, nausea/vomiting, pain, dyspnoea, constipation, appetite loss, sleeping problems, financial difficulties), one global health status/quality of life scale
		4-point Likert scale (1 – not at all to 4 – very much), except for two 7-point global health status/quality of life items
		Transformation of all scales to 0–100 scale [30]
		High scores on functional scales and global quality of life scales represent high level of functioning/quality of life
		High scores on symptom scales represent a high symptom burden
	EORTC-QLQ-MY20 [28, 29]	20-item add-on module of disease-specific symptoms and functional impact for multiple myeloma, added onto the EORTC-QLQ-C30
		Two symptom subscales (disease symptoms and side-effects of treatment), two functional subscales (body image and future perspectives)
		4-point Likert scale (1 – not at all to 4 – very much)
		Transformation of all scales to 0–100 scale
		High scores on functional scales represent high levels of functioning. High scores on symptom scales represent a high symptom burden.
	EuroQOL-5D-3L [27]	Time trade-off utility measure from a 5-item health status assessment and a visual analogue scale (generic health state outcome)
		5 items: mobility, self-care, usual activities, pain/discomfort, anxiety/depression; global health status measured by one visual-analogue scale (0–100)
		3-point Likert scale for 5 items (no problems, some/moderate problems, extreme problems)
		Five items form EQ5D Index score, transformed into health status
		Range of −0.59 to 1.0 points (higher scores indicate better health state with 1.0 representing full health), standardised according to country-specific norms (UK and US norms)

#### Sociodemographic and clinical information assessed via patient interview

Demographic information on age, gender, marital status, ethnicity, religion, educational level and occupation status was obtained directly from the patient. Performance status was assessed by applying the Eastern Cooperative Oncology Group (ECOG) scale with 0 ‘Fully active’ to 4 ‘Completely disabled’ [35].

#### Disease and treatment details extracted from medical records

Disease and clinical details were extracted from the patient’s medical notes. These were information on the date of diagnosis, the immunoglobulin type (Ig), and the clinical stage of myeloma. The International Staging System (ISS) [36] for myeloma was used to stage the disease at diagnosis on the basis of the reported β_2_-microglobulin and albumin parameters in the clinical notes. Time since diagnosis in months as a measure of disease duration was calculated by subtracting the date of the interview from the data of diagnosis. The current phase of illness was classified as newly diagnosed (pre-treatment or undergoing first-line treatment), being in a treatment-free interval (watch and wait or stable disease with no evidence of disease progression) or relapsed/progressive disease (second line therapy or above, lack of response or progression on treatment or receiving palliative care) [37].

Treatment details were also extracted from the medical records. It was recorded whether patients were currently on treatment, the types and dates of current and previous treatments and the response to these treatments [38]. From this information, a classification was derived of current and previous treatments, treatment intensity, number of lines of treatment received and whether patients were in a treatment or a treatment-free interval at the time of the survey. A treatment line was defined as any active or maintenance treatment a patient received for their myeloma disease, either as first-line treatment or after a relapse. Treatment-free intervals were intervals during which patients were classified as being in remission, receiving no active or maintenance treatment or receiving supportive treatments only (e.g. anaemia medication or bisphosphonates).

### Statistical analysis

Apart from one item (worry about sex life) on the MyPOS, missing data were less than 5 % of participants on most dependent and independent variables and tested to be missing at random. For descriptive analyses we did not impute missing values [39]. Handling of missing data in the multivariate analyses involved running a complete-case analysis as the first step und using multiple imputation in a second step [40].

Data analysis for objective (a), the description of symptom severity, palliative care concerns and HRQOL, involved determining the point prevalence with 95 % confidence intervals of MyPOS symptoms (if reported at least as ‘slight’). The *X*^*2*^-test was used for comparison of symptom burden across disease phases. The total MyPOS score (total palliative care concerns) and subscale scores of the MyPOS were compared between disease phases using univariate analysis of variance.

For objective (b), determining the factors associated with a lower quality of life and higher palliative care concerns, we used multiple linear regression models. The total MyPOS score, global quality of life scale of the QLQ-C30 and the EQ5D Index were the dependent variables and symptom and patient characteristics were independent variables. We built regression models for each outcome variable separately. Data cleaning and testing of assumptions for regression techniques (normality, skewness, kurtosis, outliers, linearity) were performed before analysis [39]. Total scores on the MyPOS, the EORTC and EQ 5D questionnaires satisfied assumptions for multivariate analysis. Multicollinearity assessment showed multicollinearity of the physical functioning subscale in the EORTC-QLQ-C30 and the “mobility” item in the MyPOS. The latter, due to its better statistical distribution, was kept in the analysis. The following strategy was used to prioritise variables for inclusion in the models: univariate linear regression models tested each of the 15 symptoms against the three outcomes. Those that were statistically significant (Bonferroni-corrected alpha level <0.003) were combined in a multivariate model that was then trimmed to exclude variables that lost significance. The initial set of clinical, treatment and demographic variables was based on a systematic review of predictors for HRQOL in multiple myeloma [41].

To test objective (c), determining whether general symptom level had a stronger influence on HRQOL than disease characteristics, we used hierarchical regression procedures. We adjusted models for each outcome variable for the influence of general symptom level (total number of symptoms on the MyPOS). Socio-demographic, disease and treatment history variables as well as HADS depression and anxiety scores found to be significant in bivariate analyses were entered into the multivariate model, which was further reduced by excluding non-significant factors.

Sample size calculations in G*Power software [42] for multiple linear regression analyses using 15 predictors, power = 80 %, α = 0.05 and a medium effect size of *F* = 0.15 for regression [43] suggested a sample size of 139, which was well exceeded in this analysis.

All analyses were conducted using SPSS 22 [44].

### Ethical issues

Research Ethics Committee approval was granted by the South East London REC-3 (ref 10/H0808/133) and by the Central London REC (13/LO/1140). Local permissions from the Research & Development departments of all 18 participating NHS hospital trusts were obtained. A complete list of participating trusts can be found in the Declarations section.

## Results

**Fig. 1:**
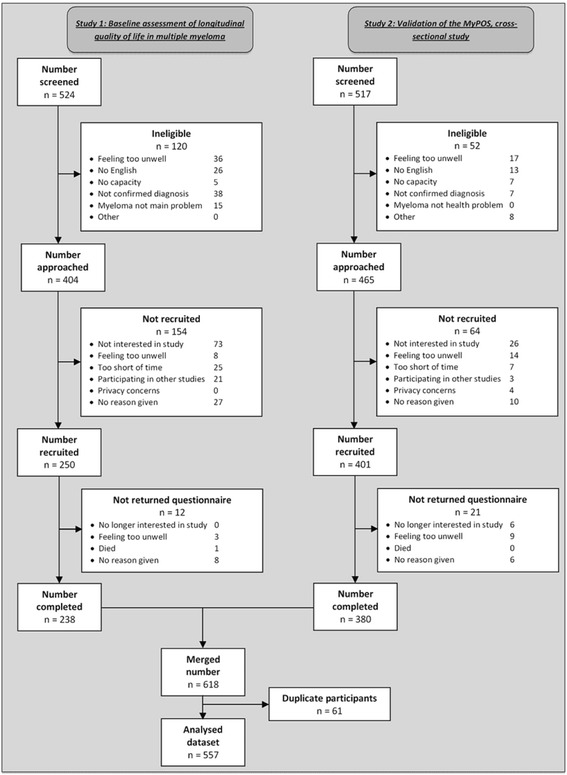
Cross-sectional analysis of symptom burden and palliative care needs in multiple myeloma: Flow chart of two study cohorts merged in analysis

Overall, 1041 patients with multiple myeloma were screened in both studies, of which 869 fulfilled the inclusion criteria and were approached. Completed questionnaires were received from 557 participants. One-hundred-seventy-two patients were ineligible for recruitment, 218 declined to participate and 33 were consented but the completed questionnaire was not received. Reasons for ineligibility and non-participation are detailed in Fig. 1.

Table 2 displays the sample characteristics of 557 myeloma patients. Their mean age was 68.4 years (SD 10.4; median: 69 years, range: 34–92 years) with a higher proportion of men taking part (61.4 %). Most participants were in a treatment-free interval; a mean 42.5 months post diagnosis; 139 (25.5 %) patients had been living with myeloma 5 years or longer. Two-hundred-fifty-eight (46.5 %) participants were currently not on active or maintenance treatment. The median number of lines of treatment received was one.

**Table 2:** Demographic and clinical characteristics of 557 patients with myeloma included in the study

	Patients	
Variable	*n*	%
a) Socio-demographic details		
Age: Mean (SD, range)	68.41	(SD 10.4; 34–92)
Gender		
Men	341	61.2
Women	209	37.5
Missing	7	1.3
Ethnicity		
White British/Irish/Other white background	513	92.1
Black African or Black Caribbean	19	3.4
Mixed ethnic background	4	0.7
Other	14	2.5
Missing	7	1.3
Marital status		
Single	44	7.9
Married	400	71.8
Divorced or separated	36	6.5
Widowed	68	12.2
Missing	9	1.6
Occupational status		
Working or student	82	14.7
Not working or retired	467	83.9
Missing	8	1.4
b) Disease factors		
Current phase of illness		
Newly diagnosed	102	18.3
Treatment-free interval/stable disease	266	47.8
Relapsed/progressive/palliative stage	182	32.7
ISS stage at diagnosis		
I	154	27.6
II	109	19.6
III	116	20.8
Missing	178	32.0
Time since diagnosis in years: Mean (SD)	3.53	(3.4)
Median, range (in years)	2.5	(0.08–23.6)
Immunoglobulin type		
IgG	314	56.4
IgA	118	21.2
Kappa or lambda light chain	95	17.1
Other	15	2.7
Missing	15	2.7
ECOG performance status		
0 Fully active	188	33.8
1 Restricted	222	39.9
2 Unable to work	87	15.6
3 or 4 – Limited selfcare/confined	50	9.0
Missing	10	1.8
Total number of symptoms on MyPOS		
0	5	0.9
1–5	175	31.4
6–8	168	30.2
9–15	205	36.8
Missing	4	0.7
d) Treatment factors		
Lines of treatment: Median (range)	1	(0–6)
Previously untreated	30	5.4
1 line received	249	44.7
2 lines received	155	27.8
3 or more lines received	123	22.1
Currently on treatment	292	52
Active chemotherapy	213	–
Undergoing autologous stem cell transplant	5	–
Maintenance therapy	74	–
Current MM treatment		
Bortezomib	59	27.6
Lenalidomide	89	41.9
Thalidomide/Pomalidomide	56	26.5
Alkalyting agent	111	51.9
Other	2	0.9
Combination chemotherapy	110	51.4
Intensity of treatments received		
None	54	9.7
Chemotherapy only	303	54.4
Chemotherapy and stem cell transplant	161	28.9
More than one transplant	32	5.7
Missing	7	1.3

*Abbreviations*: *ECOG* Eastern Cooperative Oncology Group performance status, *ISS* International staging system classification of myeloma [36], *MyPOS*: Myeloma Patient Outcome Scale, *SD* Standard deviation

### Prevalence of myeloma-specific symptoms and concerns

**Fig. 2:**
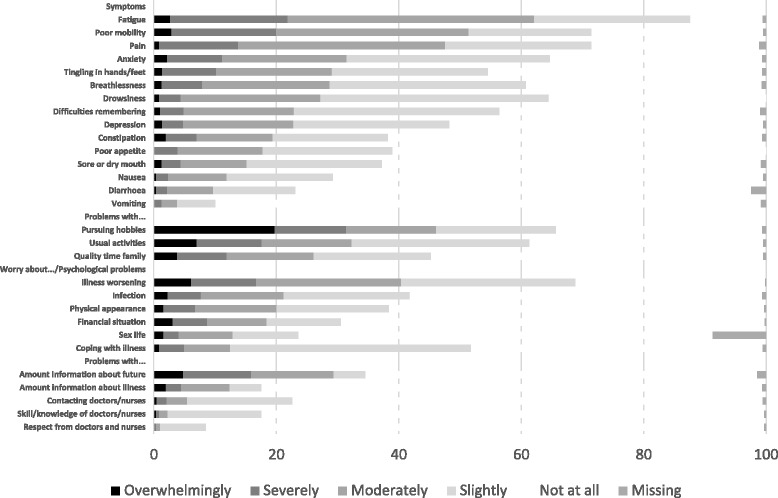
Prevalence and severity of individual symptoms and other problems as reported on the MyPOS (%) for *n* = 557 patients. Symptoms and problems in each category are listed in order of severity

Patients reported a mean of 7.2 symptoms (SD = 3.3, median: 7, range: 0–15). The most burdensome symptoms, scored as ‘severe’ or ‘overwhelming’ on the MyPOS, were fatigue (with 21.9 % scoring it as burdensome), pain (13.8 %), and tingling in the hand/feet (10.2 %) (Fig. 2 and Additional file 1: Table S1). Three symptoms were present in 60–88 % of patients - pain (71.5, 95 % CI: 67–76 %), fatigue (87.6, 95 % CI: 85–90 %) and breathlessness (60.8, 95 % CI: 57–65 %). Difficulty remembering things, tingling in the hand/feet and poor mobility were present in 50–70 % of participants. Less prevalent symptoms were constipation (38.3 %), mouth problems (sore or dry mouth, 37.3 %), anxiety (31.5 %), nausea (29.3 %), diarrhoea (23.2 %), depression (22.8 %) and vomiting (10.1 %).

The most burdensome problems and concerns existed in the domains functioning, emotional wellbeing, and information needs. These included problems with carrying out usual activities (32.3 %); worrying that the illness might get worse (40.4 %), and not having enough information about what might happen in the future (29.4 %). The mean total MyPOS score was 21.5 (SD = 13.4), indicating a moderate level of concerns.

### Symptoms and concerns per treatment phase

The prevalence and severity of symptoms differed according to disease phase. Of the three groups – newly diagnosed, treatment-free interval, and relapsed/progressive disease - those with relapsed/progressive disease had the highest mean number of symptoms (*M* = 5.91, SD = 2.63; versus *M* = 4.91 in the newly diagnosed group and *M* = 4.77 in a treatment-free interval). On the symptom level, differences between disease phases were found for shortness of breath (*X*^*2*^: 12.5, *p* = 0.002), constipation (*X*^*2*^: 8.1, *p* = 0.018), mouth problems (*X*^*2*^: 9.98, *p* = 0.007), and tingling in the hands and feet (*X*^*2*^: 18.93, *p* < 0.001) with more patients in the relapsed/progressive phases of disease suffering from these symptoms than expected (Table 3).

**Table 3:** Outcome data scores for total sample and comparison of symptoms and palliative care needs across disease phases

	Score			Newly diagnosed (*n* = 102)			Stable (*n* = 268)			Progressive, relapsed stage (*n* = 184)			Test	
Measure	*n*	Mean, SD	Median (range)	*n*	Mean, SD	Median (range)	*n*	Mean, SD	Median (range)	*n*	Mean, SD	Median (range)	*F* value	*p*
Time since diagnosis (months)	552	42.3 (40.7)	29.9 (0.1–283)	102	10.4 (16.8)	4.6 (0.2–103.1)	267	44.2 (39.8)	30.4 (0.49–239.9)	183	57.3 (41.8)	57.3 (41.8)	52.2	0.001*
ECOG Performance status	551	–	1 (0–4)	101	–	1 (0–3)	268	–	1 (0–4)	182	–	1 (0–4)	*X* ^*2*^: 24.4	**0.002**
MyPOS^a^														
Total score	468	21.5 (13.5)	19 (0–61)	86	22.9 (13.4)	20 (1–61)	229	18.9 (13.1)	17 (1–59)	150	24.7 (13.4)	23 (0–61)	9.6	**0.001**
Symptoms and function	526	76.2 (16.6)	78.8 (30.4–100)	96	75.8 (14.5)	76.8 (36–100)	253	79.1 (14.3)	80.4 (30.4–100)	175	72.2 (14.8)	71.4 (34–100)	11.9	**0.001**
Emotion and coping	499	80 (16.6)	84.4 (18.8–100)	94	77.1 (17.2)	81.3 (34–100)	244	82.4 (16.2)	87.5 (18.8–100)	158	77.9 (16.4)	81.3 (34–100)	5.3	**0.005**
Healthcare support and information needs	544	90.8 (12.7)	95 (40–100)	99	91.2 (12.8)	95 (40–100)	264	91.1 (12.9)	100 (40–100)	178	89.8 (12.5)	95 (50–100)	0.6	0.532
EORTC-QLQ-C30^b^														
Global health status	555	61.2 (22.3)	66.7 (0–100)	102	59.5 (20.5)	66.7 (0–100)	267	65.8 (21.8)	66.7 (0–100)	183	55.2 (22.7)	50 (0–100)	12.9	**0.001**
Physical function	554	61.5 (22.5)	60 (0–100)	101	61.2 (26.7)	66.7 (0–100)	266	65.3 (25.2)	66.7 (0–100)	184	56.2 (24.6)	53.3 (0–100)	6.9	**0.001**
Role function	553	59 (33.1)	66.7 (0–100)	101	55.4 (35.6)	66.7 (0–100)	266	64.9 (30.9)	66.7 (0–100)	183	52.3 (33.5)	50 (0–100)	8.9	**0.001**
Emotional function	555	76.2 (22.1)	83.3 (0–100)	102	74.5 (23.7)	83.3 (0–100)	267	77.3 (21.3)	83.3 (0–100)	183	75.3 (22.3)	75 (0–100)	0.8	0.459
Cognitive function	555	79 (21.9)	83.3 (0–100)	102	78.1 (21.9)	83.3 (0–100)	267	81.2 (20.5)	83.3 (16.7–100)	183	76.3 (23.7)	83.3 (0–100)	2.8	0.060
Social function	554	65.1 (31.5)	66.7 (0–100)	102	60.5 (34.8)	66.7 (0–100)	267	70.2 (29.3)	66.7 (0–100)	182	60.1 (31.7)	66.7 (0–100)	7.1	**0.001**
EORTC QLQ-MY20^c^														
Disease symptoms	549	73.9 (21.2)	77.8 (0–100)	101	75.7 (20.9)	77.8 (0–100)	262	74 (20.9)	77.8 (5.6–100)	183	72.7 (21.8)	77.8 (0–100)	0.6	0.530
Side-effects of treatment	542	81.4 (14.4)	83.3 (0–100)	100	80.3 (14)	83.3 (43–100)	261	83.5 (14)	86.7 (30–100)	178	78.8 (14.9)	80 (23–100)	6.1	**0.002**
Body image	551	77.9 (30.5)	100 (0–100)	100	79 (31.7)	100 (0–100)	265	79.6 (28.2)	100 (0–100)	183	74.9 (32.8)	100 (0–100)	1.4	0.247
Future perspective	549	64.6 (26.5)	66.7 (0–100)	100	61.4 (28.1)	66.7 (0–100)	264	67.2 (25.1)	77.8 (0–100)	182	62.1 (27.3)	66.7 (0–100)	2.8	0.061
EuroQOL-5D-3L														
EQ5D Index score	550	0.65 (0.28)	0.69 (−0.5–1)	101	0.66 (0.28)	0.69 (−0.18–1)	264	0.67 (0.27)	0.69 (−0.18–1)	182	0.59 (0.29)	0.69 (−0.35–1)	4.5	**0.012**
EQ5D Visual analogue scale VAS	318	63.51 (20.02)	61 (0.5–100)	68	58.8 (19.8)	60 (0.5–96)	139	69 (19.6)	69.5 (11–100)	111	59.5 (19.1)	60 (10–100)	9.82	**0.001**

^a^MyPOS: Myeloma Patient Outcome Scale: comprises 27 items, higher scores indicate higher symptom burden/more palliative care needs, MyPOS subscale scores transformed to 0–100 scale to allow for comparison to subscale scores from the EORTC QLQ-C30 and –MY20 questionnaires

^b^EORTC QLQ-C30: For the EORTC-QLQ-C30, higher scores on functioning subscales and the global quality of life scale indicate better functioning/better quality of life

^c^EORTC-QLQ-MY20: For the myeloma module of the EORTC quality of life questionnaire higher scores indicate more problems/symptoms in subscales

*Bold values denote significant p-values (>0.05)

**Fig. 3:**
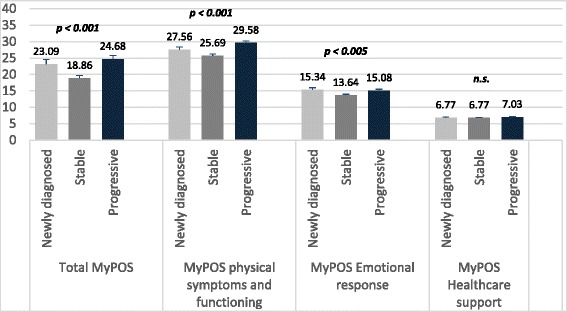
Differences in the total MyPOS and MyPOS subscales in three phases of myeloma disease

Similarly, patients with relapsed/progressive disease had the highest mean total MyPOS score (*M* = 24.68), followed by newly diagnosed patients (*M* = 23.1) and patients in a treatment-free interval (*M* = 18.8). On the subscale level, univariate analysis of variance showed that differences exist in Functioning/Symptoms (*F* = 11.919, *p* = 0.001) and the Emotional response subscale (*F* = 5.36, *p* = 0.005) between the phases with post-hoc tests indicating that patients with relapsed and progressive disease have more problems in these areas than those in the stable phases of myeloma (Fig. 3).

**Fig. 4:**
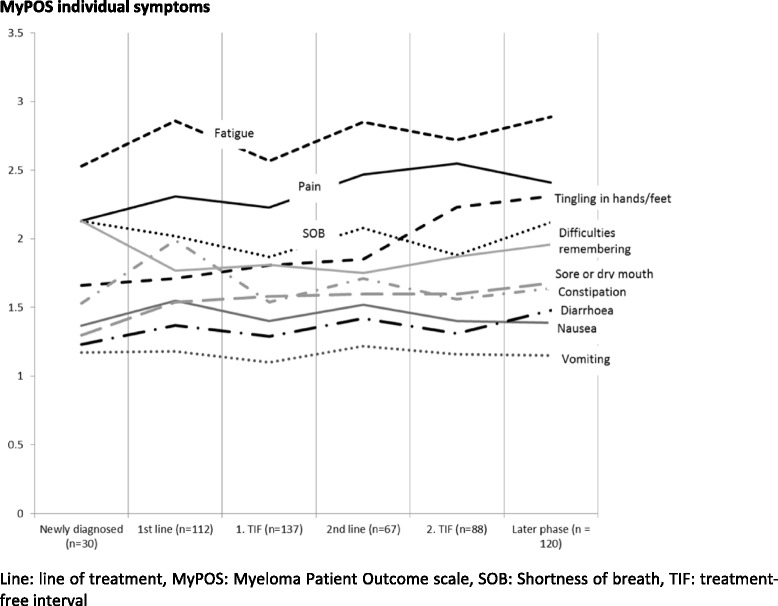
Mean MyPOS symptoms and subscale scores per treatment phase. A higher score indicates a higher symptom burden in the individual symptom items. *Line* line of treatment, *MyPOS* Myeloma Patient Outcome scale, *SOB* Shortness of breath, *TIF* treatment-free interval

A more fine-grained analysis of phase according to treatment (number of treatment lines or treatment-free interval), shown in Fig. 4, was conducted to better understand the differences in symptom burden and problems according to phase. The symptoms fatigue, pain and shortness of breath showed a high severity throughout all treatment phases, with the latter being overtaken by tingling in the hands and feet as the third most severe symptom from the second treatment-free interval onwards. Scores for all symptoms tended to be higher during treatment-intervals than in treatment-free intervals. Scores for sore or dry mouth, diarrhoea, tingling in the hands/feet, shortness of breath and difficulties remembering were highest for those participants in the later phases of disease.

### Factors associated with myeloma-specific problems and concerns

All symptoms and functioning scales of the EORTC QLQ-C30 were significantly associated in bivariate analyses with the total MyPOS score. The only demographic characteristic being associated with high palliative care concerns was age (see Additional file 1: Tables S2 and S3). Clinical characteristics that were significantly different for those in the lower vs higher half of the MyPOS total score distribution were phase of illness (with a higher proportion of newly diagnosed and relapsed patients reporting a higher MyPOS total score), receiving treatment, an ECOG performance status of 2 and general symptom level. In the first multivariate model and after adjusting for general symptom level, only the symptoms fatigue, pain, anxiety, dry mouth and the physical function and social function subscales remained significantly independently associated with the outcome. The final parsimonious multivariable model with demographic and clinical factors showed significant associations of general symptom level, pain, anxiety, dry mouth, physical function, age, and being either in the newly diagnosed or relapsed/progressive disease phase with high palliative care concerns (see Table 4).

**Table 4:** Regression models for outcome variables a) palliative care concerns (total MyPOS score), b) global quality of life (EORTCQLQ-C30 subscale), and c) generic health-related quality of life (EQ5D Index score) and their association with demographic, clinical characteristics and symptom burden (*n* = 557)

	Palliative care concerns			Global quality of life QLQ-C30			EQ5D Index		
Independent variables	Coefficient	Lower CI	Upper CI	Coefficient	Lower CI	Upper CI	Coefficient	Lower CI	Upper CI
(Constant)	20.639**	14.189	27.089	105.788**	100.986	110.589	1.168**	1.093	1.242
General symptom level	1.439**	1.136	1.741	–	–	–	–	–	–
Pain	0.046*	0.016	0.076	−2.572*	−4.168	−0.975	−0.100**	−0.129	−0.071
Weakness/lack of energy	–	–	–	−5.741**	−7.395	−4.086	–	–	–
Drowsiness	–	–	–	–	–	–	0.032	0.000	0.064
Dry mouth	1.395**	0.558	2.233	–	–	–	–	–	–
HADS Anxiety	1.100**	0.891	1.308	−2.519*	−4.091	−0.947	–	–	–
HADS Depression	–	–	–	−3.749**	−5.446	−2.052	−0.075**	−0.102	−0.048
Age	−0.136**	−0.204	−0.069	–	–	–	–	–	–
Being in the stable/plateau phase^a^	−2.693**	−4.096	−1.290	4.804**	2.127	7.482	–	–	–
ECOG performance status 2^b^	–	–	–	−3.654	−7.449	0.141	–	–	–
ECOG performance status 3/4 – limited self-care or completely disabled^c^	–	–	–	–	–	–	−0.159**	−0.248	−0.070
Physical function/Poor mobility	−0.138**	−0.174	−0.101	−5.085**	−6.610	−3.560	−0.082**	−0.110	−0.054
Adjusted R^2^	0.879			0.514			0.584		
*F*, *P*	*F* = 192.205	*P* < 0.001		*F* = 85.522	*P* < 0.001		*F* = 61.924	*P* < 0.001	

*ECOG* Eastern Cooperative Oncology Group performance status, *CI* confidence interval, *EQ5D* EuroQol-5D-3L, *HADS* Hospital Anxiety and Depression Scale, *MyPOS* Myeloma Patient Outcome Scale

^a^Reference group is patients being unstable, i.e. newly diagnosed or having relapsed, progressive or palliative disease or being in a treatment-interval

^b^Reference group is ECOG performance status of 0

^c^Reference group is ECOG performance status of 0

*p < 0.05

**p < 0.001

### Multiple regression analysis of quality of life

In the first multivariate model including all symptoms and demographic and clinical characteristics found positively associated in the bivariate analyses (see Additional file 1: Tables S2 and S3), only the symptoms pain, fatigue, anxiety and depression as well as poor mobility remained significant in their association to the global quality of life item of the QLQ-C30. Controlling for general symptom level did not alter the results. In the final multivariate trimmed model, the quality of life score was significantly and independently associated with a higher pain level, higher fatigue level, more mobility problems, more anxiety and depression and an ECOG performance status of 2 (Table 4). Those in a treatment-free interval experienced better quality of life. The final multivariate trimmed model for the outcome EQ5D Index score contained the variables pain, drowsiness, poor mobility, depression and ECOG performance status of 2, but no effect of phase of illness was found, nor an effect of general symptom level (Table 4).

## Discussion

This is the first study to compare levels of symptom burden and quality of life problems among patients at different stages of the disease. We found a persistently high symptom burden, even during treatment-free phases of disease in multiple myeloma, and providing evidence for the association and potential mediation of general symptom level, pain, fatigue, mental health and physical function on disease-related problems and HRQOL.

The findings demonstrate the persistently high symptom burden and compromise in HRQOL, expressed by a high mean number of symptoms, with pain, fatigue, symptoms of peripheral neuropathy and breathlessness as the most commonly reported symptoms. These persist into the later stages of myeloma, thereby confirming results from the Nordic Myeloma Study group [10] and from the Eindhoven Profiles registry [11] regarding the high symptom burden and the importance of pain, fatigue and breathlessness, together with symptoms of peripheral neuropathy, in myeloma. Our analysis expands these findings by showing that symptoms may well extend and remain a burden in the treatment-free intervals. A similar persistent high prevalence of pain, neuropathic and other, and fatigue was observed by Boland and co-authors in a sample of patients with multiply relapsed but stable disease [23]. A lower HRQOL in global and subdomains of the EORTC QLQ-C-30 and MY-20 measures was also described in a cross-sectional study by Acaster et al. [6], suggesting persistent symptom burden. However, it should be borne in mind that these findings come from cross-sectional studies. Longitudinal observational evidence in myeloma is rare, with the few studies not using secondary analysis of RCT data enrolling patients at the stage directly pre or post first-line treatment and not focusing on advanced stages [17–22].

One surprising finding was the high prevalence of breathlessness that was reported by 60.8 % of participants. The severity of shortness of breath had a mean of close or above 2.0 in all treatment phases – from diagnosis and prior to first-line treatment to later phases post the second treatment-free interval. However, this finding might be explained by cardiac or pulmonary complications either resulting from the disease itself, from treatments received (with patients receiving immunomodulatory agents or bortezomib being at increased risk of experiencing pulmonary adverse events) [45, 46], or – given that our study included a predominantly older population with a mean age of 68.4 years – also being a consequence of age-related comorbidities [47]. A limitation of our study is that number of comorbidities was not assessed and that lack of assessing this potential confounder might affect the relationship between symptoms, performance status and palliative care concerns or quality of life. Future studies should focus on the relationship between comorbidity, treatment intensity, disease progression and HRQOL [48].

To better understand which patients with multiple myeloma would profit from targeted supportive care interventions, a regression analysis of associations between patient, disease, treatment characteristics and palliative care concerns or quality of life was conducted. Multiple myeloma, despite being an incurable disease with patients ultimately dying from it, its related complications, or from the side effects of treatment, is still not recognised as a disease that warrants palliative care involvement [49]. This is mainly due to the disease, like many other haematological cancers, not following a linear trajectory of progression in which the end of life is well-defined. Rather, the progression is interspersed with intermittent periods of remission and stable disease, relapse, multiple lines of treatment, the potential for sudden deterioration and death due to disease- or treatment-related complications and patients continuing to receive and to respond to treatment even in advanced disease [50–52]. Our finding of a persistently high symptom burden, even during treatment-free intervals, shows that decisions regarding involvement of palliative care to support and help with the impact on quality of life cannot be based on clinical response to treatment as this will miss a substantial number of patients who would benefit from additional supportive or palliative care services [53, 54]. This matters because research on needs in general cancer and myeloma has shown that those patients with high unmet needs, low quality of life and a high symptom burden are at increased risk of shortened survival [55–57].

Results from regression analyses support other authors’ findings of patient’s self-reporting of symptoms providing independent prognostic information [8, 55, 57]. However, contrary to our hypothesis, we found that general symptom level did not act as a mediator in the hierarchical regression analyses for all outcomes. This means it is not the high number of symptoms that might indicate who is in need of supportive care, but specific symptoms might better serve that purpose. Especially the presence of pain or fatigue indicates burden and is associated with high palliative care concerns; results that are similar to findings by Kripp et al. [54]. In our study, general symptom level did not remain independently associated with global quality of life when sociodemographic and clinical variables were entered into the regression analysis, contrary to a recent study using a different outcome measure to determine general symptom level [12]. This might be because Jordan et al. [12] did not take into account variables like functioning. Our findings from the regression analyses support the hypothesised relationships between symptoms, function, emotional response and quality of life in the Wilson and Cleary model [58].

Contrary to the study of predictors for survival in myeloma [38], clinical variables such as ISS stage, myeloma subtype or treatment-related variables did not remain significantly independently associated with quality of life in our study. Other factors, such as low performance status and pain, do overlap. Depression and anxiety were significant factors for all three outcomes. The importance and persistence of mental health problems have been demonstrated in other studies, also as predictors for survival [ 55, 57]. Overall, this points towards patients needing more support in all phases and that focusing resources on the end of life, which is hard to define in multiple myeloma, or clinical response criteria misses a potentially large number of patients who experience a high burden of disease- and treatment-related problems. Early integration of palliative care, alongside monitoring of HRQOL and symptoms, could help targeting supportive care services towards those in need and might help better symptom management. These approaches have shown to be valuable in monitoring treatment adverse events in haematology [59, 60].

Our study had several limitations. Despite the high response rate, this was a cross-sectional study with non-random sampling. Selection bias might limit the validity of the findings. Although many of the screened and eligible patients for the study took part and we aimed to recruit a consecutive sample, there was some non-response. Among the reasons for declining to take part “feeling too unwell” or considering the study “too burdensome” were most frequently named, suggesting that those who declined might have had more symptoms and concerns and also might have had more difficulties coping with the consequences of myeloma and its treatment. However, this did not hinder us to recruit a substantial number of patients with relapsed or progressive disease (32.7 %). The high number of patients during treatment-free intervals and with stable disease points towards the fact that the results from this study under-represent the views of those that might have a shorter and more acute disease trajectory and more severe symptoms. Prevalence estimates for symptoms might therefore be biased towards under-estimation.

The majority of patients were recruited from tertiary cancer centres, although we tried to obtain a mix of recruiting centres. More patients were sampled from out-patient than from in-patient clinics. This led to an under-representation of patients receiving stem cell transplant at the time of the study (5 %) and might have imbalanced the sample towards those with higher functional performance status (only 9 % of patients had an ECOG performance status of 3 or 4). A diverse patient group was included with diverse treatment histories which makes it difficult to distinguish between disease symptoms and treatment-related toxicities. A further limitation of our study is the lack of collecting information on co-morbidities for patients. This information was only available for a part of the sample and could not be obtained validly from all medical notes. We are aware that it is therefore not possible to understand this potential confounding factor.

This study uses a cross-sectional design. Therefore, independent variables in the regression analyses and any correlation reported represent association but no prediction. Moreover, this study did not follow patients as they naturally progressed through different phases of disease. Comparison between phases therefore relies on comparison between different patients. Physiological variables like haemoglobin, albumin or other variables that indicate disease activity were not extracted from the medical records and could not be considered in the regression analysis.

## Conclusions

This study showed the importance of regular assessment of symptom burden and of quality of life in routine clinical care. The current practice of one single holistic needs assessment potentially misses periods of persistently high problems, specifically pain, fatigue, breathlessness but also mental health problems that occur during the advanced stages and even during treatment-free intervals. The early integration of supportive and palliative services for those experiencing high physical and emotional symptoms could help improve symptom management and therefore help maintain or optimise patient’s quality of life. Focusing on traditional parameters to monitor the disease progression might not help identify those patients with myeloma that experience a low quality of life.

## Abbreviations

CI, confidence interval; ECOG, Eastern Cooperative Oncology Group; EORTC QLQ-C30, European Organization for Research and Treatment of Cancer (EORTC) quality of life questionnaire C30; EORTC QLQ-MY20, European Organization for Research and Treatment of Cancer (EORTC) myeloma-specific quality of life questionnaire MY20; EQ5D, EuroQOL group health status questionnaire 5D-3L; HADS, Hospital Anxiety and Depression Scale; HRQOL, health-related quality of life; Ig, immunoglobulin type; ISS, International Staging System; M, mean; MyPOS, Myeloma Patient Outcome Scale; n.s., non-significant; SD, standard deviation; SOB, shortness of breath; TIF, treatment-free interval; UK, United Kingdom; US, United States of America

## Additional file

Additional file 1: Figure S1. Myeloma Patient Outcome Scale (MyPOS). All questions are preceded by “Over the past week…”. **Table S1.** Prevalence and severity of myeloma-specific symptoms and problems (MyPOS) in 557 multiple myeloma patients. **Table S2.** Univariate associations of symptoms with EORTC QLQ –global quality of life scale, EQ5D index and visual analogue (VAS) scale scores and the Myeloma Patient Outcome Scale total score, using linear regression with bootstrapping (1000 samples). **Table S3.** Bivariate associations of independent variables with the the outcomes a) MyPOS total palliative care concerns, b) EQ5D Index, d) Global health status (EORTC QLQ-C30), *n* = 557. (DOCX 42 kb)
