# Supplementary material for: The impact of disease-related symptoms and palliative care concerns on health-related quality of life in multiple myeloma: a multi-centre study
Source: BMC Cancer. 2016 Jul 7;16:427. doi: 10.1186/s12885-016-2410-2 (PMC4937527; doi:10.1186/s12885-016-2410-2)
Supplement: Additional file 1: Figure S1. — Myeloma Patient Outcome Scale (MyPOS). All questions are preceded by “Over the past week…”. Table S1. Prevalence and severity of myeloma-specific symptoms and problems (MyPOS) in 557 multiple myeloma patients. Table S2. Univariate associations of symptoms with EORTC QLQ –global quality of life scale, EQ5D index and visual analogue (VAS) scale scores and the Myeloma Patient Outcome Scale total score, using linear regression with bootstrapping (1000 samples). Table S3. Bivariate associations of independent variables with the the outcomes a) MyPOS total palliative care concerns, b) EQ5D Index, d) Global health status (EORTC QLQ-C30), n = 557. (DOCX 42 kb) [file 12885_2016_2410_MOESM1_ESM.docx]

**Additional file 1**

**Figure S1**. Myeloma Patient Outcome Scale (MyPOS). All questions are preceded by “Over the past week…”.

| **1** | What are your main problems or concerns at the moment? | [Open question with three empty boxes for respondent to complete, numbered 1-3] | | | | |
| --- | --- | --- | --- | --- | --- | --- |
| **2** | Below is a list of symptoms, which you may or may not have experienced. For each symptom please tick one box that best describes how it has affected you over the past week: | Not at all | Slightly | Moderately | Severely | Over-whelmingly |
|  |  | I have not had this symptom in the past week | Little or no effect on activities or concen-tration | Some effect on activities or concen-tration | Marked effect on activities or concen-tration | Unable to think of anything  else |
| **a** | Pain |  |  |  |  |  |
| **b** | Fatigue or lack of energy |  |  |  |  |  |
| **c** | Shortness of breath |  |  |  |  |  |
| **d** | Diarrhoea |  |  |  |  |  |
| **e** | Constipation |  |  |  |  |  |
| **f** | Nausea (feeling like you are going to be sick) |  |  |  |  |  |
| **g** | Vomiting (being sick) |  |  |  |  |  |
| **h** | Mouth problems |  |  |  |  |  |
| **i** | Poor mobility |  |  |  |  |  |
| **j** | Tingling in the hands and / or feet |  |  |  |  |  |
| **k** | Difficulty remembering things |  |  |  |  |  |
| **l** | Please list any other symptoms not mentioned above, and tick one box to show how they have affected you over the past week: | [Three boxes beneath symptoms list for respondent to add additional symptoms, numbered 1-3] | | | | |
| **3** | Have you been able to carry out your usual activities without help from others? | Yes, as much as I wanted | Most of the time | Sometimes | Occasionally | No, not at all |
| **4** | Have you been able to pursue your hobbies and leisure activities? |  |  |  |  |  |
| **5** | Have you been able to spend quality time with family and friends? |  |  |  |  |  |
| **6** | Have you been worrying about your sex life? | No, not at all | Occasionally | Sometimes | Most of the time | Yes, always |
| **7** | Have you been feeling depressed? |  |  |  |  |  |
| **8** | Have you been feeling anxious or worried about your illness or treatment? |  |  |  |  |  |
| **9** | Have you been worrying about infections? |  |  |  |  |  |
| **10** | Have you been worrying about your physical appearance? |  |  |  |  |  |
| **11** | Have you been worrying about your financial situation? |  |  |  |  |  |
| **12** | Have you been worrying that your illness will get worse? |  |  |  |  |  |
| **13** | Have you felt able to cope with your illness and treatment? | Yes, always | Most of the time | Sometimes | Occasionally | No, not at all |
| **14** | Are you able to contact your doctors or nurses for advice if needed? |  |  |  |  |  |
| **15** | Do your doctors and nurses show a good standard of knowledge and skill when treating you? |  |  |  |  |  |
| **16** | Do your doctors and nurses show care and respect when treating you? |  |  |  |  |  |
| **17** | Do you have enough information about your illness and treatment? | Enough Information | Information received | Information received | Very little information | No information received |
| **18** | Do you have enough information about what might happen to you in the future? | the right amount for me | but hard to understand | but would like more | and would like more | and would like information |
| **19** | How did you complete this questionnaire? | On my own | With help from a friend or relative | With help from a staff member |  |  |

**Table S1.** Prevalence and severity of myeloma-specific symptoms and problems (MyPOS) in 557 multiple myeloma patients

|  |  |  | Prevalence | 95% CI | Not at all (0) | | Slight (1) or Moderate (2) | | Severe (3) or Overwhelming (4) | | Missing data |
| --- | --- | --- | --- | --- | --- | --- | --- | --- | --- | --- | --- |
|  | M | SD | n (%) |  | n | % | n | % | n | % | % |
| ***Symptoms*** |  |  |  |  |  |  |  |  |  |  |  |
| Pain | 1.35 | 1.05 | 398 (71.5%) | 67-76% | 152 | 27.3 | 321 | 57.7 | 77 | 13.8 | 1.2 |
| Breathlessness | 0.99 | 0.99 | 339 (60.9%) | 57-65% | 214 | 38.4 | 295 | 52.9 | 44 | 7.9 | 0.8 |
| Fatigue | 1.75 | 0.99 | 488 (87.6%) | 85-90% | 66 | 11.8 | 366 | 65.7 | 122 | 21.9 | 0.6 |
| Nausea | 0.44 | 0.78 | 163 (29.3%) | 25-33% | 391 | 70.2 | 150 | 26.9 | 13 | 2.4 | 0.5 |
| Vomiting | 0.15 | 0.51 | 56 (10.1%) | 7-13% | 496 | 89.0 | 49 | 8.8 | 7 | 1.3 | 0.9 |
| Poor appetite | 0.61 | 0.88 | 218 (39%) | 35-43% | 337 | 60.5 | 196 | 35.1 | 22 | 3.9 | 0.5 |
| Constipation | 0.67 | 1.00 | 213 (38.3%) | 34-42% | 340 | 61.0 | 174 | 31.3 | 39 | 7.0 | 0.7 |
| Sore or dry mouth | 0.58 | 0.89 | 207 (37.3%) | 33-41% | 344 | 61.8 | 183 | 32.9 | 24 | 4.4 | 1.1 |
| Drowsiness | 0.97 | 0.89 | 353 (63.4%) | 59-67% | 198 | 35.5 | 330 | 59.2 | 23 | 4.2 | 1.1 |
| Poor mobility | 1.46 | 1.15 | 398 (71.5%) | 68-75% | 156 | 28.0 | 287 | 51.5 | 111 | 20.0 | 0.5 |
| Diarrhoea | 1.46 | 1.15 | 129 (23.2%) | 20-27% | 414 | 74.3 | 117 | 21 | 12 | 2.2 | 2.5 |
| Tingling in hands/feet | 0.96 | 1.06 | 304 (54.6%) | 50-59% | 249 | 44.7 | 247 | 44.4 | 57 | 10.2 | 0.7 |
| Difficulties remembering | 0.86 | 0.92 | 314 (56.5%) | 52-61% | 237 | 42.5 | 287 | 51.6 | 27 | 4.9 | 1.1 |
| ***Problems with/ Worry about...*** | |  |  |  |  |  |  |  |  |  |  |
| Usual activities | 1.2 | 1.2 | 342 (61.4%) | 57-66% | 212 | 38.1 | 244 | 43.8 | 98 | 17.6 | 0.5 |
| Pursuing hobbies | 1.64 | 1.5 | 366 (65.7%) | 62-70% | 187 | 33.6 | 191 | 34.3 | 175 | 31.4 | 0.7 |
| Spending quality time with family/friends | 0.87 | 1.16 | 252 (45.3%) | 41-50% | 302 | 54.2 | 186 | 33.4 | 66 | 11.9 | 0.5 |
| Sharing feelings with family | 0.89 | 1.2 | 245 (45%) | 41-49% | 304 | 54.5 | 168 | 31.1 | 77 | 13.9 | 1.5 |
| Sex life | 0.46 | 0.9 | 132 (23.7%) | 20-28% | 376 | 67.5 | 109 | 19.6 | 23 | 4.1 | 8.8 |
| Feeling depressed | 0.78 | 0.96 | 269 (48.3%) | 44-53% | 285 | 51.2 | 242 | 43.5 | 27 | 4.8 | 0.5 |
| Feeling at peace | 2.38 | 1.14 | 439 (78.9%) | 76-82% | 110 | 19.8 | 345 | 62.1 | 94 | 16.8 | 1.3 |
| Anxious about illness/treatment | 1.1 | 1.05 | 360 (64.7%) | 60-69% | 193 | 34.6 | 298 | 53.5 | 62 | 11.2 | 0.7 |
| Family anxious/worried about patient | 1.23 | 1.14 | 369 (66.1%) | 62-70% | 179 | 32.2 | 284 | 50.9 | 85 | 15.2 | 1.7 |
| Infection | 0.74 | 1.04 | 233 (41.8%) | 38-46% | 320 | 57.5 | 190 | 34.1 | 43 | 7.7 | 0.7 |
| Physical appearance | 0.67 | 1.00 | 214 (38.4%) | 34-43% | 341 | 61.2 | 176 | 31.6 | 38 | 6.8 | 0.4 |
| Financial situation | 0.61 | 1.07 | 170 (30.6%) | 28-35% | 385 | 69.1 | 122 | 21.9 | 48 | 8.7 | 0.4 |
| Illness worsening | 1.32 | 1.19 | 384 (68.9%) | 65-73% | 172 | 30.9 | 291 | 52.2 | 93 | 16.7 | 0.2 |
| Coping with illness/treatment | 0.71 | 0.85 | 289 (51.8%) | 48-56% | 265 | 47.6 | 261 | 46.8 | 28 | 5.0 | 0.5 |
| Contacting doctors/nurses | 0.31 | 0.67 | 127 (22.7%) | 19-27% | 427 | 76.7 | 115 | 20.6 | 12 | 2.1 | 0.5 |
| Skill/Knowledge of doctors/nurses | 0.21 | 0.53 | 98 (17.6%) | 15-21% | 457 | 82.0 | 93 | 16.7 | 5 | 0.9 | 0.4 |
| Respect from doctors and nurses | 0.10 | 0.39 | 48 (8.6%) | 6-11% | 507 | 91.0 | 46 | 8.2 | 2 | 0.4 | 0.4 |
| Having enough information about illness/treatment | 0.37 | 0.89 | 98 (17.6%) | 15-21% | 455 | 81.7 | 73 | 13.1 | 25 | 4.5 | 0.7 |
| Information about what might happen in the future | 0.86 | 1.29 | 193 (34.6%) | 31-39% | 356 | 63.9 | 104 | 18.7 | 89 | 15.9 | 1.4 |
| Addressing practical matters resulting from illness | 0.5 | 0.97 | 142 (25.5%) | 22-29% | 405 | 72.8 | 106 | 19.0 | 36 | 6.5 | 1.7 |
| MyPOS Total score | 21.5 | 13.4 | range: 0-61 |  |  |  |  |  |  |  |  |

**Table S2. Univariate associations of symptoms with EORTC QLQ –global quality of life scale, EQ5D index and visual analogue (VAS) scale scores and the Myeloma Patient Outcome Scale total score, using linear regression with bootstrapping (1000 samples)**

|  | **QL2** | | **EQ5D index** | | **EQ5D VAS** | | **MyPOS total score** | |
| --- | --- | --- | --- | --- | --- | --- | --- | --- |
| **Variable** | **Coefficient**  **(95% CI)** | ***p*** | **Coefficient**  **(95% CI)** | ***p*** | **Coefficient**  **(95% CI)** | ***p*** | **Coefficient**  **(95% CI)** | ***p*** |
| **Pain** | -10.48  (-12.12, -8.92) | **<0.001** | -0.17  (-0.19, -0.15) | **<0.001** | -8.90  (-10.72, -7.08) | **<0.001** | 8.07  (7.17, 8.97) | **<0.001** |
| **Shortness of breath** | -8.23  (-10.13, -6.42) | **<0.001** | -0.08  (-0.10, -0.06) | **<0.001** | -6.42  (-8.50, -4.35) | **<0.001** | 6.32  (5.22, 7.43) | **<0.001** |
| **Weakness** | -12.85  (-14.35, -11.18) | **<0.001** | -0.12  (-0.14, -0.10) | **<0.001** | -10.47  (-12.39, -8.56) | **<0.001** | 8.73  (7.78, 9.69) | **<0.001** |
| **Nausea** | -8.75  (-11.13, -6.65) | **<0.001** | -0.11  (-0.14, -0.08) | **<0.001** | -6.21  (-8.79, -3.63) | **<0.001** | 9.10  (7.78, 10.42) | **<0.001** |
| **Vomiting** | -6.66  (-9,86, -3.80) | **<0.001** | -0.07  (-0.13, -0.03) | 0.002 | -2.96  (-7.34, 1.43) | 0.186 | 8.98  (6.80, 11.16) | **<0.001** |
| **Poor appetite** | -9,57  (-12.82, -5.86) | **<0.001** | -0.11  (-0.16, -0.07) | **<0.001** | n/a | n/a | 8.13  (6.33, 9.92) | **<0.001** |
| **Constipation** | -5.02  (-6.88, -3.37) | **<0.001** | -0.07  (-0.10, -0.04) | **<0.001** | -3.65  (-5.86, -1.43) | **0.001** | 5.25  (4.14, 6.35) | **<0.001** |
| **Sore or dry mouth** | -6.03  (-8.08, -3.90) | **<0.001** | -0.09  (-0.11, -0.06) | **<0.001** | -4.20  (-6.72, -1.67) | **0.001** | 6.24  (4.99, 7.50) | **<0.001** |
| **Drowsiness** | -10.21  (-13.20, -7.05) | **<0.001** | -0.10  (-0.13, -0.06) | **<0.001** | n/a | n/a | 7.73  (5.98, 9.48) | **<0.001** |
| **Poor mobility** | -11.28  (-12.62, -9.95) | **<0.001** | -0.15  (-0.17, -0.14) | **<0.001** | -9.99  (-11.49, -8.51) | **<0.001** | 7.63  (6.84, 8.43) | **<0.001** |
| **Diarrhoea** | -6.35  (-9,04, -3.52) | **<0.001** | -0.07  (-0.10, -0.03) | **<0.001** | -4.59  (-7.54, -1.65) | 0.002 | 5.85  (4.29, 7.40) | **<0.001** |
| **Tingling** | -6.22  (-7.99, -4.52) | **<0.001** | -0.06  (-0.08, -0.04) | **<0.001** | -5.68  (-7.62, -3.74) | **<0.001** | 4.22  (3.14, 5.29) | **<0.001** |
| **Difficulty remembering** | -7.41  (-9.30, -5.70) | **<0.001** | -0.09  (-0.11, -0.06) | **<0.001** | -4.74  (-7.00, -2.48) | **<0.001** | 6.54  (5.26, 7.64) | **<0.001** |
| **Anxiety** | -9.56  (-11.27, -7.83) | **<0.001** | -0.12  (-0.14, -0.10) | **<0.001** | -8.89  (-10.79, -6.99) | **<0.001** | 8.92  (8.13, 9.64) | **<0.001** |
| **Depression** | -10.34  (-12.15, -8.59) | **<0.001** | -0.14  (-0.17, -0.12) | **<0.001** | -8.26  (-10.50, -6.01) | **<0.001** | 9.25  (8.24, 10.25) | **<0.001** |
| **EORTC Physical function** | - |  | - |  | - |  | -0.37  (-0.41, -0.34) | **<0.001** |
| **EORTC Role Function** | - |  | - |  | - |  | -0.29  (-0.31, -0.26) | **<0.001** |
| **EORTC Social function** | - |  | - |  | - |  | -0.30  (-0.33, -0.27) | **<0.001** |

**Table S3. Bivariate associations of independent variables with the the outcomes a) MyPOS total palliative care concerns, b) EQ5D Index, d) Global health status (EORTC QLQ-C30), n = 557**

| N (%) | ***High palliative care concerns*** | ***Low palliative care concerns*** | **p-value** | ***Low EQ5D Index*** | ***High EQ5D Index*** | **p-value** | ***Low global QOL*** | ***High global QOL*** | **p-value** |
| --- | --- | --- | --- | --- | --- | --- | --- | --- | --- |
| **Sociodemographic details** |  |  |  |  |  |  |  |  |  |
| ***Age (years)***  Mean (SD) | 66.2 (11.4) | 69.4 (9.8) | **0.001** | 67.7 (10.6) | 69.1 (10.3) | 0.109 | 68.4 (10.8) | 68.2 (9.8) | 0.892 |
| ***Gender*** |  |  | 0.441 |  |  | 0.029 |  |  | 0.129 |
| Men | 142 (30.3%) | 151 (32.3%) |  | 180 (32.7%) | 158 (28.7%) |  | 230 (41.4%) | 113 (20.4%) |  |
| Women | 91 (19.4%) | 83 (17.7%) |  | 136 (24.7%) | 74 (13.5%) |  | 142 (25.6%) | 68 (12.3%) |  |
| ***Ethnicity*** |  |  | 0.035 |  |  | 0.456 |  |  | 0.170 |
| White background | 211 (45.3%) | 222 (47.6%) |  | 296 (54.1%) | 215 (39.3%) |  | 343 (62.1%) | 172 (31.2%) |  |
| Non-white or mixed | 22 (4.7%) | 11 (2.4%) |  | 20 (3.7%) | 16 (2.9%) |  | 28 (5.1%) | 9 (1.6%) |  |
| ***Marital status*** |  |  | 0.188 |  |  | 0.061 |  |  | 0.037 |
| Married | 166 (35.3%) | 171 (36.5%) |  | 220 (40%) | 174 (31.6%) |  | 263 (47.4%) | 136 (24.5%) |  |
| Single, divorced, widowed | 67 (14.3%) | 61 (13%) |  | 96 (17.5%) | 55 (10%) |  | 108 (19.5%) | 43 (7.7%) |  |
| ***Occupational status*** |  |  | 0.049 |  |  | **<0.001** |  |  | **<0.001** |
| Working | 30 (6.4%) | 44 (9.4%) |  | 31 (5.7%) | 51 (9.3%) |  | 39 (7.1%) | 43 (7.8%) |  |
| Not working | 203 (43.6%) | 189 (40.6%) |  | 285 (52.2%) | 179 (32.8%) |  | 332 (60.3%) | 137 (24.9%) |  |
| **Disease factors** |  |  |  |  |  |  |  |  |  |
| ***Phase of illness*** |  |  | **<0.001** |  |  | 0.136 |  |  | **<0.001** |
| Newly diagnosed | 47 (10.1%) | 40 (8.6%) |  | 55 (10.1%) | 46 (8.4%) |  | 75 (13.6%) | 27 (4.9%) |  |
| Stable phase | 94 (20.2%) | 135 (29%) |  | 145 (26.5%) | 119 (21.8%) |  | 158 (28.6%) | 109 (19.7%) |  |
| Relapsed/progressive | 92 (19.7%) | 58 (12.4%) |  | 116 (21.2%) | 66 (12.1%) |  | 138 (25%) | 45 (8.2%) |  |
| ***ISS stage at diagnosis*** |  |  | 0.266 |  |  | 0.965 |  |  | 0.163 |
| ISS stage I | 68 (21%) | 66 (20.4%) |  | 87 (23%) | 66 (17.4%) |  | 99 (26%) | 54 (14.2%) |  |
| ISS stage II | 41 (12.7%) | 54 (16.7%) |  | 62 (16.4%) | 46 (12.1%) |  | 66 (17.3%) | 43 (11.3%) |  |
| ISS stage III | 52 (16%) | 43 (13.3%) |  | 69 (18.2%) | 49 (12.9%) |  | 86 (22.6%) | 33 (8.7%) |  |
| ***MM disease duration***  Mean (SD) | 41.7 (43) | 41.8 (38.1) | 0.977 | 41.9 (40.6) | 42.6 (40.9) | 0.842 | 40.7 (40.5) | 45.8 (41.3) | 0.196 |
| ***Type of myeloma*** |  |  | 0.233 |  |  | 0.087 |  |  | 0.084 |
| Light chain | 43 (9.7%) | 37 (8.3%) |  | 60 (11.5%) | 33 (6.3%) |  | 69 (13.1%) | 26 (5%) |  |
| IgG or IgA | 177 (39.8%) | 188 (42.2%) |  | 240 (46.2%) | 187 (36%) |  | 278 (53%) | 152 (29%) |  |
| **Treatment factors** |  |  |  |  |  |  |  |  |  |
| ***Receiving treatment*** |  |  | **<0.001** |  |  | 0.462 |  |  | **<0.001** |
| Yes | 140 (30%) | 103 (22.1%) |  | 168 (30.7%) | 121 (22.1%) |  | 216 (39.1%) | 74 (13.4%) |  |
| No | 93 (20%) | 130 (27.9%) |  | 148 (27.1%) | 110 (20.1%) |  | 155 (28.1%) | 107 (19.4%) |  |
| ***Lines of treatment***  Median (IQR) | 1 (1 – 2) | 2 (1 – 3) | 0.015 | 2 (1-2) | 1 (1-2) | 0.291 | 2 (1 – 2) | 1 (1 – 2) | 0.072 |
| ***Treatment phase*** |  |  | 0.014 |  |  | 0.239 |  |  | 0.004 |
| Newly diagnosed | 9 (1.9%) | 17 (3.6%) |  | 13 (2.4%) | 16 (2.9%) |  | 15 (2.7%) | 15 (2.7%) |  |
| First line treatment | 54 (11.6%) | 39 (8.4%) |  | 67 (12.2%) | 45 (8.2%) |  | 85 (15.4%) | 27 (4.9%) |  |
| First treatment-free interval | 46 (9.9%) | 73 (15.7%) |  | 72 (13.2%) | 62 (11.3%) |  | 78 (14.1%) | 59 (10.7%) |  |
| Second line treatment | 28 (6%) | 25 (5.4%) |  | 38 (6.9%) | 29 (5.3%) |  | 50 (9.1%) | 16 (2.9%) |  |
| Second treatment-free interval | 34 (7.3%) | 35 (7.5%) |  | 59 (10.8%) | 28 (5.1%) |  | 62 (11.2%) | 26 (4.7%) |  |
| Later phase | 62 (13.3%) | 44 (9.4%) |  | 67 (12.2%) | 51 (9.3%) |  | 81 (21.8%) | 38 (6.9%) |  |
| **Functional and symptom status** |  |  |  |  |  |  |  |  |  |
| ***ECOG performance status*** |  |  | **<0.001** |  |  | **<0.001** |  |  | **<0.001** |
| 0 – Fully active | 51 (11.1%) | 111 (24.1%) |  | 59 (10.9%) | 126 (23.3%) |  | 81 (14.9%) | 107 (19.6%) |  |
| 1 – Restricted | 84 (18.3%) | 103 (22.4%) |  | 134 (24.8%) | 84 (15.6%) |  | 157 (28.8%) | 64 (11.7%) |  |
| 2 – Unable to work | 56 (12.2%) | 12 (2.6%) |  | 74 (13.7%) | 13 (2.4%) |  | 81 (14.9%) | 5 (0.9%) |  |
| 3/4 – Limited self care, confined to bed | 38 (8.3%) | 5 (1.1%) |  | 45 (8.3%) | 5 (0.9%) |  | 46 (8.4%) | 4 (0.7%) |  |
| ***Number of symptoms*** |  |  | **<0.001** |  |  | **<0.001** |  |  | **<0.001** |
| 0 | 0 | 5 (1.1%) |  | 0 | 5 (0.9%) |  | 0 | 5 (0.9%) |  |
| 1-5 | 13 (2.8%) | 135 (28.9%) |  | 37 (6.8%) | 135 (24.7%) |  | 53 (9.6%) | 122 (22.1%) |  |
| 6-8 | 67 (14.3%) | 74 (15.8%) |  | 109 (20%) | 58 (10.6%) |  | 127 (23%) | 39 (7.1%) |  |
| 9-15 | 152 (32.5%) | 21 (4.5%) |  | 170 (31.1%) | 32 (5.9%) |  | 191 (34.7%) | 14 (2.5%) |  |

Bonferroni correction used per outcome variable, critical alpha level <0.003

ECOG: Eastern Cooperative Oncology Group performance status, IQR: Interquartile range, ISS: International Staging system for multiple myeloma [24], SD: standard deviation
